# Application of machine learning algorithms for prediction of abortion and its determinants among women of reproductive-age in East African countries

**DOI:** 10.1038/s41598-026-54541-w

**Published:** 2026-05-27

**Authors:** Biruktawit Lelisa Eticha, Geleta Nenko Dube, Sefefe Birhanu Tizie, Tesfaye Deribe Bedada, Smegnew Gichew Wondie, Selamawit Gashaw Teshome, Andualem Addisu Birlie, Muluken Belachew Mengistie, Ayana Alebachew Muluneh, Gelgelo Wodessa, Nega Abebe Meshesha

**Affiliations:** 1 Department of Health informatics, School of Public Health, College of Medicine and Health Sciences, Wachemo University, Hossana, Ethiopia; 2 School of Public Health, Institute of Health, Bule Hora University, Bule Hora, Ethiopia; 3 Department of Health Informatics, College of Medicine and Health Science, Debre Markos University, Debre Markos, Ethiopia; 4 Department of Public Health, College of Medicine and Health Science, Mizan Tepi University, Mizan Aman, Ethiopia; 5 School of Public Health, Health Science Campus, Debre Berhan University, Debre Berhan, Ethiopia; 6 Department of Health Informatics, College of Medicine and Health Science, Samara University, Samara, Ethiopia; 7 Department of Health Informatics, College of Medicine and Health Science, Wollo University, Dessie, Ethiopia; 8 Department of Public Health, Institute of Health, Dambi Dollo University, Dambi Dollo, Ethiopia

**Keywords:** Demographic and health survey, East Africa, Machine learning, Abortion, Diseases, Health care, Medical research, Risk factors

## Abstract

In low- and middle-income nations, abortion ranks among the top five causes of maternal mortality. It is associated to a pregnancy and childbirth-related complications. Despite this, there are few scientific researches focusing on predicting abortion and its determinants in East African countries. Therefore, this study aimed to predict abortion and its determinants among women of reproductive-age in East African countries. Community-based cross-sectional study design was used from eleven East African countries DHS dataset spanning 2015 to 2023. The study participants were all reproductive age women who were selected using a two-stage stratified sampling technique. The machine-learning algorithms were applied to predict abortion and its determinants using Python, particularly Jupiter notebook in Google colab. Data cleaning, one-hot encoding, data splitting, and ten-fold cross-validation were performed. Ten machine learning algorithms and SHAP were used to select and interpret the best model. From the total of 372,053 reproductive age women in East Africa, 12.2% participants perform abortion. Random forest was found the best model for training data with 91% of an AUC and 86% of accuracy. According to SHAPE analysis, women who have been never in union, women whose age 15–19 years, women whose age 20–24 years, women from Ethiopia, and women who not used any method were the top four features of abortion. This study identified that random forest classifier was emerged as the best-performing model to predict abortion among women reproductive age in East African countries. Marital status, marital status, age, country, Contraceptive use by method type, and living children plus current pregnancy were key determinant of abortion in East African countries. Governments and health systems should provide access to comprehensive family planning services, reproductive health education, and maternal health support.

## Introduction

Abortion is a serious health problem that causes many complications, maternal fatalities, and large expenses associated with people, families, and healthcare systems^1–3^.

Approximately 73 million induced abortions are performed globally each year, of which 45% are identified as unsafe^3,4^. Approximately 97% of unsafe abortions occur in developing countries. Abortion is responsible for about 8% of maternal fatalities globally^1^. About seven million women in the developing world, or 6.9 per 1000 women between the ages of 15 and 44, received care in 2012 for problems caused on by unsafe abortion procedures^2^. Sub-Saharan Africa (SSA) had an estimated yearly rate of 33 abortions per 1000 women aged 15 to 49 between 2015 and 2019, which was higher than rates in developed nations and showed no difference among its four subregions^5^. About 620,300 induced abortions were performed in Ethiopia in 2014, translating to an annual abortion rate of 28 per 1000 women between the ages of 15 and 49. Of these, about 294,100 took place outside of medical institutions^6^.

Pregnancy and delivery-related complications claim the lives of many women worldwide; low- and middle-income nations account for roughly 99.0% of maternal deaths; Pregnancy and delivery-related complications claim the lives of many women worldwide; low- and middle-income nations account for roughly 99.0% of maternal deaths^7^. Abortion can happen on its own or on intentionally; the latter is known as induced abortion, and it can be either safe or risky. Abortion is extremely harmful and can result in complications like bleeding, infection, and uterine perforatio^8^.

Approximately 210 million women worldwide become pregnant every year. Unwanted pregnancies account for 80 million of them. Of these pregnancies, 46 million are aborted, and 19 million result in unsafe abortions^9,10^. 13% of maternal fatalities worldwide are related to abortion, particularly unsafe abortions; this relates to 37 deaths per 100,000 live births in Sub-Sharan Africa (SSA) and 12 deaths per 100,000 in South Asia^11^.

Previous research conducted worldwide has shown that abortion is associated with a number of factors. Teenagers and women over 35 are more likely to have an abortion^12,13^, not attending formal education^14,15^, residents^16,17^, poor individuals^18^, single women^19,20^, rural residence^21^.

pervious delivery by cesarean Sect.^22^, low parity^13^, maternal under-nutrition^23^, use of substance like cigarette and tobacco^20,24–26^, and multiple pregnancies^27–29^, currently working^20^, traditional family planning methods^20^, and media exposure^20^.

This is one of the benefits of including multinational abortion data in this analysis. It serves as a guide to the plans and interventions of various international, continental and national organizations to enable them which region is severely affected and needs urgent further research and policy amendments^30,31^.Despite abortion being associated with pregnancy and birth-related complications and the vital cause of maternal mortality, especially in low- and middle-income countries^20^, to the best of our knowledge, there is no pooled data that predict abortion and its determinants of in East African countries that is the region where low- and middle-income countries founded. Therefore, this study aimed to predict abortion and its determinants among reproductive-aged women in East African countries. Conducting this study will help decide maternal health based on the best available scientific evidence.

## Materials and methods

### Study area, design, and period

This study was conducted in East African countries using the DHS dataset, representing eleven nations. Geographically, East Africa is a sub-region of Africa that includes nineteen internationally known countries (Burundi, Comoros, Djibouti, Ethiopia, Eritrea, Kenya, Madagascar, Malawi, Mauritius, Mozambique, Rwanda, Seychelles, Somalia, Tanzania, Uganda, Zambia, South Sudan, Zimbabwe, and Sudan)^32^, of these nineteen East African countries, 14 have DHS data, whereas Five do not (Djibouti, Somalia, South Sudan, Seychelles, and Mauritius). Among these 14 countries, one has restricted DHS data (Eritrea), and two (Comoros and Sudan) has old data set from 1989 to 2012. Thus, this study included 11 countries (Burundi, Ethiopia, Uganda, Rwanda, Tanzania, Mozambique, Madagascar, Zimbabwe, Kenya, Zambia, and Malawi) of recent standard DHS data collected between 2015 and 2023 to make more representatives for East Africa. This study used a nationally representative dataset; collected using a community-based cross-sectional study design. The current study was conducted between February 01, 2025 and June 31, 2025 using demographic and health surveys that were conducted between 2015 and 2023 GC in East African countries.

### Data source

This study used measured DHS program data, including the Women’s Record (IR file) that could be accessed online. The datasets from selected East Africa countries were extracted from the official DHS program database, which can be accessed at https://dhsprogram.com/data/available-datasets.cfm.We. The DHS Program has collected representative data on nutrition, HIV, population, and health through standardized surveys in more than 90 countries^33^. Demographic and Health Surveys are nationally representative household surveys that gather data on FP and other issues in low and middle-income nations and the surveys are carried out by universities or local government organizations with technical support from ICF via the DHS program^34^.

### Source and study population

All 15–49 aged women living in East African countries were source population and all women aged 15–49 years and included for the surveys conducted in the respective countries in East Africa were study population of this study.

### Eligibility criteria

All women aged 15–49 living in East African countries who were either permanent residents of the selected households or visitors who stayed in the household the night before the survey that conducted between the years of 2015 to 2023GC were included in the study. However, all women aged 15–49 living in East African countries with missing data on the outcome variable were excluded from the study.

### Sample size determination

The sample was limited to responses from women who were aged 15–49 during the survey. Thus, the analysis was restricted to a sample of 372,053 women.

### Sampling technique and procedure

DHS uses two-stage stratified cluster sampling approach: (1) determining enumeration areas with a probability proportionate to size and power distribution, modifying to satisfy the DHS survey’s minimum cluster requirement per survey domain and (2) selecting the households in EAs^35^. All women aged 15–49 who were either permanent residents of the selected households or visitors who stayed in the household the night before the survey were eligible to be interviewed using a standardized woman’s questionnaire. This uniformity in procedures ensured comparability of data across countries, thus enabling a pooled data approach to examine the abortion and associated factors among reproductive age women in East African countries.

### Study variables

In this study, the main outcome variable was abortion. The independent variables were socio-demographic and economic related factors (age, residence, educational level, wealth index, marital status, currently working, distance from health facility, smokes cigarette, a mobile telephone ownership, exposure to mass media, and country), and sexual and reproductive health related factors (current pregnancy status, contraceptive use by method type, knowledge of any method, living children plus current pregnancy, and current breastfeeding status). The selection of these independent variables was based on a comprehensive review of previous literature.

### Operational definitions

Exposure to mass media information: women considered to be exposed if, within the last few months, they received information either through mobile text messaging, print media (such as magazines and newspapers), radio, or television^36^.

### Data collection procedures

The data for this study was draw from nationally representative DHS data conducted in elven (i.e. Burundi (DHS 2016GC), Ethiopia (DHS 2016GC), Kenya (DHS 2022GC), Madagascar (DHS 2021GC), Malawi (DHS 2017GC), Mozambique (DHS 2022-2023GC), Rwanda (DHS 2019-2020GC), Tanzania (DHS 2022GC), Uganda (DHS 2018GC), Zambia (DHS 2018GC) and Zimbabwe (DHS 2015GC)) countries in East Africa.

DHS gathers a variety of objective and self-reported data, with a particular emphasis on indicators of mortality, nutrition, mother and child health, fertility, reproductive health, and adult self-reported health behaviors. Standardized data collection methods across nations, consistent content over time, strong response rates, nationwide coverage, and excellent interviewer training are some of the DHS’s main advantages. For the nation’s population, it established nationally representative samples; it routinely collected every five years using structured methodologies pretested and validated tools and it follows the same standard sampling procedure, questionnaires, data collection, and coding which is internationally led by the DHS program^37^. This makes multi-country analysis simple and reasonable.

### Data quality assurance

To ensure data quality, missing data was addressed systematically using STATA software, employing appropriate techniques such as imputation or deletion based on the extent and nature of the missing values. Variables were re-categorized to reduce multicollinearity, ensuring more robust statistical analyses and improving model interpretability. Once the data cleaning and transformation processes are complete, the dataset was exported to Excel and saved as CSV file format for further analysis.

### Data analysis procedures

The general framework in the available literature was based on Yufeng Guo’s 7 Steps of Machine Learning for predicting abortion. The framework describes the seven steps of supervised machine learning, which are summarized below: Data collection, preparation, model selection, training, evaluation, parameter adjustment, and prediction^38,39^.

In this study, descriptive statistics were employed to describe socio-demographic and economic, and sexual and reproductive health related characteristics in terms of frequency and percentage. A machine learning algorithm was conducted using Python and analyzed on Google Colab^40,41^, utilizing secondary data from the DHS (2015–2023) of elven East African countries.

### Data preprocessing/preparation

The first step in machine learning is data pre-processing, which entails changing or encoding the data to ensure it can be interpreted by a computer^42^. A continual improvement strategy for models in the machine learning pipeline was used. This process included selecting and engineering relevant features, splitting the data, model training, and model evaluation, model optimization, choosing the top performer model, and deploying the selected model for prediction. Data cleaning feature engineering and data splitting are among the data preparation techniques that were used in this study.

### Data cleaning

Data cleaning includes addressing missing values, identifying and eliminating outliers, and dealing with imbalanced outcome variable categories^43^. To handle missing values, the K-nearest neighbor imputation technique was utilized^39^. This imputation technique was chosen for its high reliability and sensitivity in imputation of missing values than other methods^38^. Various visualization techniques and statistical methods to identify outliers were employed. Additionally, multicollinearity was assessed by examining the correlation matrix and considering a correlation value above 0.8 between two pairs of variables as indicative of high correlation^44^.

### Feature selection/engineering

Feature engineering is the process of finding, obtaining, and altering the most pertinent features from the available data to create machine learning models that are more precise and effective; It was the approach of extracting the required beneficial factors from a dataset that can enhance the accuracy of machine learning predictions^45^.

A dataset with redundant, irrelevant, and inconsistent features may be lowering model performance and it is crucial to apply feature selection methods to improve the accuracy of model performance while reducing training time and over fitting^46^. Furthermore, it is a rather efficient strategy for reducing model complexity and improving the processing of machine learning algorithms^47^. In this study, one-hot encoding to encode nominal categorical variables and label encoding for ordinal categorical variables^48^ were employed.

### Data balancing

A common challenge in data mining and machine learning is data imbalance, which frequently results in worse classification accuracy for instances that belong to the minority class^49^. To tackle this issue SMOTE was applied.

To avoid ML models biased towards the majority class in this study, the training data was balanced using SMOTE, and a random oversampling approach. SMOTE generates synthetic examples (new observations) that resemble the minority class by interpolating between minority class samples in the feature space, rather than precise duplicates of existing examples. However, overlapping or resemblance between synthetic and original samples is unavoidable even after oversampling^50^.

### Model selection and development

In this study, the dependent variable, abortion, required a classification approach, as it was divided into “Yes” and “No” categories. To make predictions, selection of appropriate classifiers was needed.

To evaluate the predictive capabilities of machine learning algorithms for predicting abortion, eleven algorithms (Dummy classifier, Random forest, Decision tree, KNN, XGboost, Logistic regression, Naïve bayes, Light boost, ANN, Adaboost, and Catboost) were employed. Each algorithm has its unique approach and characteristics. These algorithms were chosen based on how well they perform classification tasks and how well they mesh with the features of this dataset^51,52^.

### Model training

In this particular study, a straightforward approach was employed; where the data was divide into an 80% training set and a 20% testing set. Though there are other methods, that provide a less biased or positive estimate of model competence, the well-known k-fold cross-validation method was employed during this study since it is straightforward to learn: This division makes it possible to evaluate each predictive model’s performance efficiently^53^. The models were trained using a tenfold cross-validation technique on the training data. Ten-fold separates all observations into ten equal-sized sample groups or folds. Nine folds are used to train the model, and one fold is then used for testing ten times. Therefore, the average of the values calculated in the loop is the ten-fold cross-validation performance metric.

### Model evaluation

In machine learning, model training and evaluation are crucial for building a reliable predictive model^54^. After model training, the model’s performances are reviewed and compared against one another. One popular technique for evaluating the effectiveness of a classification model is the confusion matrix, which is a straightforward cross-tabulation of the actual and predicted categories for the outcome variable. The performance criteria, which include the overall accuracy, precision, recall, and F1 score and were employed in this study to evaluate the effectiveness of the selected classifiers, can be calculated using confusion metrics. Additionally, the performance of the ML models was assessed using receiver operating characteristic (ROC) curves, and the value 0.5 = no discrimination. 0.5–0.7 = Poor discrimination. 0.7–0.8 = Acceptable discrimination. 0.8–0.9 = Excellent discrimination^55^.

AUC is a popular and powerful performance statistic for analyzing binary classifier performance and determining the model’s predictive classification capacity. A range of 0.5 indicated little capacity for prediction, and 1.0 indicated extraordinary or flawless predictive ability^56^.

The confusion matrix, which uses the model’s output as a binary class, is a standard performance measurement tool used in machine learning classification tasks; the confusion matrix was crucial for this study to determine the recall, precision, f1-score, and performance metrics of accuracy.

### Hyperparameter tuning

Initially, all models were trained using the scikit-learn package’s default hyperparameters. The optimal model’s hyperparameters were adjusted using the Optuna framework following model selection^57^. By defining hyperparameter optimization as the process of minimizing or maximizing an objective function (like accuracy), Optuna accepts a set of hyperparameters as input and employs the Bayesian framework to better understand the probability of optimal values and to avoid unnecessary computation for the combination of non-performing parameters during the search. Traditional hyperparameter tuning methods like grid search and randomized search, which both take explicitly user-defined hyperparameters and optimize the model solely by those hyperparameters, are less effective and flexible than this framework^43^.

### Prediction

In this study, abortion was determined based on top predictors identified during the analysis. Hence, whether a woman was abort or not was determined through the best-performing classifier with a specified accuracy.

### Model interpretation/explanation using SHAP

**Fig. 1:**
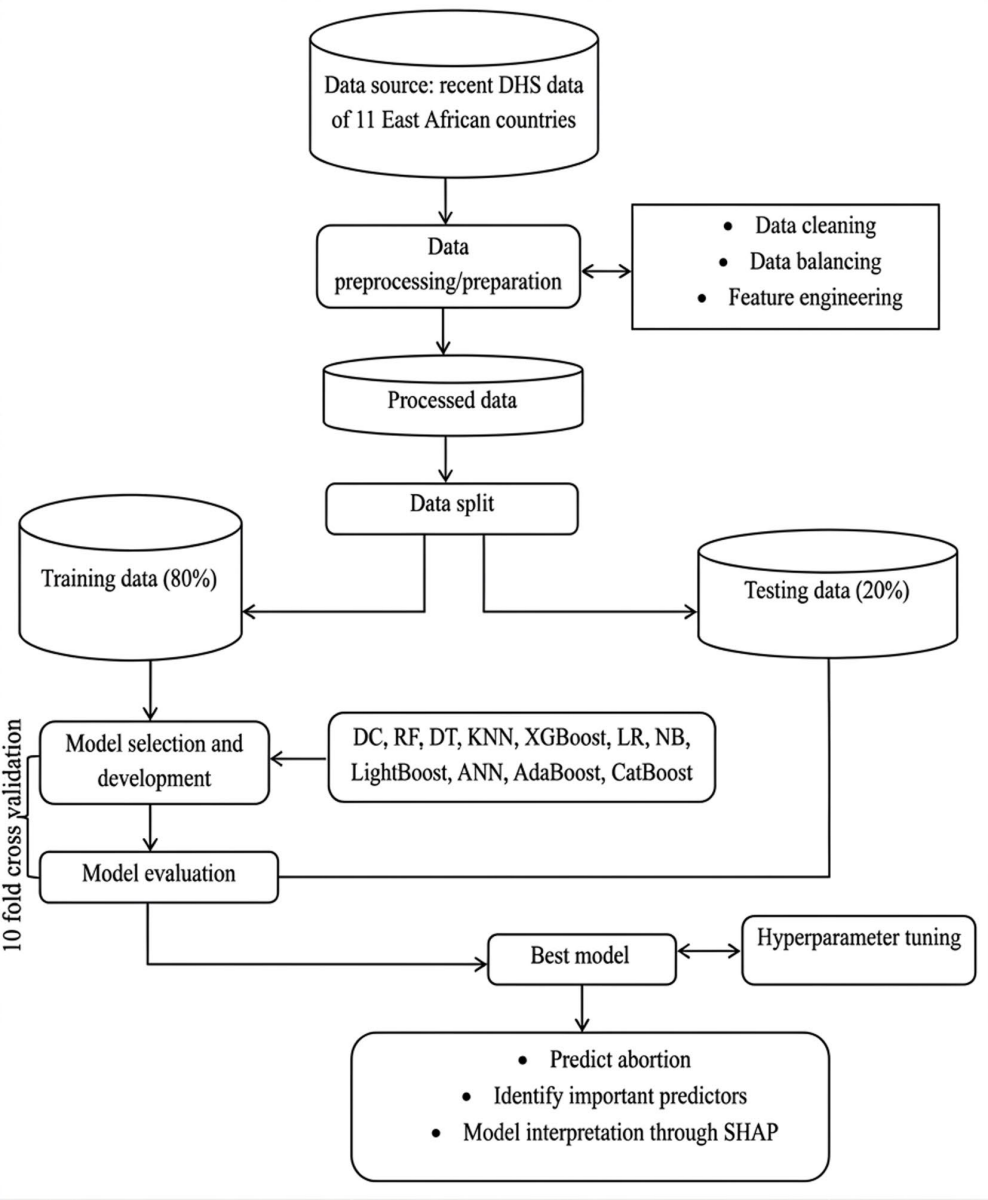
Flowchart of data preparation and analysis for predicting abortion. DC=Dummy Classifier; RF=Random Forest; DT= Decision Tree; KNN = K- nearest Neighbors; XGboost= eXtreme Gradient Boosting; LR=Logistic Regression; NB= Naive Bayes; Lboost=Light boost; ANN= Artificial Neural Network.

SHAP analysis uses game theory to evaluate and explain any machine learning model’s predictions, either locally or globally^58^. The SHAP analysis’s basic idea is to calculate each predictor’s marginal contribution to the outcome variable prediction result. For this study, SHAP analysis was applied for two purposes:

1. SHAP global interpretability provides a unified measure of feature relevance based on Shapley values, which are calculated by calculating the contribution of each feature to the prediction and aggregating it across the whole population. Several studies used SHAP as a feature selection mechanism, and their results show that machine learning using the SHAP value feature selection approach performs better in classification and has a higher capacity for model explanation^59–61^.
2. To plot the aggregate Shapley value of that specific feature for each sample to assess the impact of each predictor on abortion^61^ (Fig. 1).

## Results

### Socio-demographic and economic characteristics of the study participants

In this study a total number of 372,053 reproductive age women were included. The descriptive statistics revealed details about reproductive age women across different characteristics. In terms of age distribution, the largest group, comprising 81,771 (22.0%), fell into the 15 to 19 age range. Regarding residence, 256,314 (68.9%) lived in rural areas and 115,739 (31.1%) in urban areas. A total of 215,461 (57.9%) of women reported being currently working, while 156,592 (42.1%) indicated they were not engaged on any form of work at the time of the survey. Regarding educational status, 168,888 (45.4%) were completing primary educations, while only a small percentage 21,829 (5.9%) having no formal education.

The data encompass multiple countries with Malawi 49,069 (13.2%) being the most represented while, Zimbabwe 19,880 (5.3%) had smallest proportions in the dataset (Table 1).

**Table 1:** Socio-demographic and economic characteristics of the study participants in East African countries, 2015-2023GC (*N* = 372,053).

Variables	Categories	Frequency (n)	Percent (%)
Age in years	15–19	81,771	22.0
	20–24	70,689	19.0
	25–29	60,576	16.3
	30–34	52,118	14.0
	35–39	45,201	12.1
	40–44	34,543	9.3
	45–49	27,155	7.3
Residence	Urban	115,739	31.1
	Rural	256,314	68.9
Educational level	No education	64,314	17.3
	Primary education	168,888	45.4
	Secondary education	117,022	31.5
	Higher education	21,829	5.9
Wealth index	Poorest	70,598	19.0
	Poorer	64,572	17.4
	Middle	67,885	18.2
	Richer	74,618	20.1
	Richest	94,380	25.4
Marital status	Never in union	106,808	28.7
	Married	175,066	47.1
	Living with partner	45,945	12.3
	Widowed	11,015	3.0
	Divorced	12,504	3.4
	Separated	20,715	5.6
Currently working	Yes	215,461	57.9
	No	156,592	42.1
Exposure to mass media	Yes	167,276	45.0
	No	204,777	55.0
A mobile telephone ownership	Yes	177,396	47.7
	No	194,657	52.3
Distance from health facility	Not big problem	128,869	34.6
	Big problem	243,184	65.4
Smokes cigarrettes	Yes	2,830	0.8
	No	369,223	99.2
Country	Burundi	34,533	9.3
	Ethiopia	31,359	8.4
	Kenya	49,045	13.2
	Madagascar	37,735	10.1
	Malawi	49,069	13.2
	Mozambique	26,334	7.1
	Rwanda	29,263	7.9
	Tanzania	30,483	8.2
	Uganda	36,988	9.9
	Zambia	27,364	7.4
	Zimbabwe	19,880	5.3

### Sexual and reproductive health related characteristics of the study participants

From the Sexual and reproductive health related perspective, pregnancy status was limited 27,143 (7.3%), and 243,590 (65.5%) were not use any contraceptives at the time of the survey. As shown in Table 2 below, among reproductive age women, 99,961 (26.9%) had no living children. 280,210 (75.3%) of reproductive age women in selected East African countries were not on the breastfeeding at the time of survey (Table 2).

**Table 2:** Sexual and reproductive health related characteristics of participants in East African countries, 2015-2023GC (*N* = 372,053).

Variables	Categories	Frequency (n)	Percent (%)
Current pregnancy status	Yes	27,143	7.3
	No	344,910	92.7
Contraceptive use by method type	No method	243,590	65.5
	Folkloric method	6,62	0.2
	Traditional method	9,599	2.6
	Modern method	118,202	31.8
Current breastfeeding status	Yes	91,843	24.7
	No	280,210	75.3
Knowledge of any method	Knows no method	9,260	2.5
	Knows only folkloric method	46	0.01
	Knows only traditional method	2,86	0.1
	Knows modern method	362,461	97.4
Living children plus current pregnancy	0	99,961	26.9
	1	58,799	15.8
	2	56,724	15.2
	3	48,829	13.1
	4	37,917	10.2
	5	27,608	7.4
	6+	42,215	11.3

**Fig. 2:**
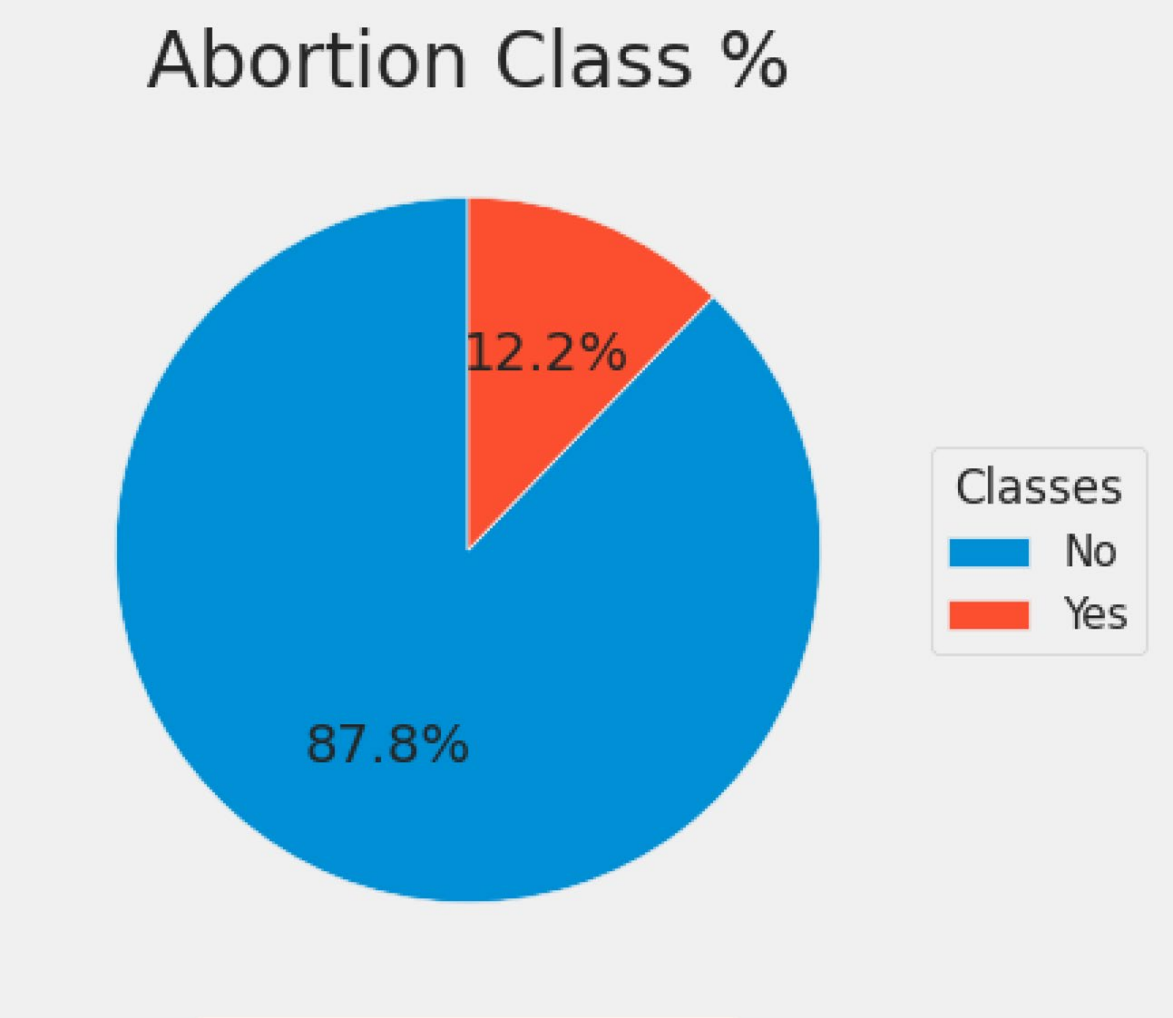
Distribution of abortion among women of reproductive age in East African countries, 2015-2023GC.

The pie chart presents the overall proportion of abortion among women of reproductive age across East African countries. The data was categorized in to two groups; women not aborted (shown in blue) and women aborted (shown in red). A total of 87.8% of women reported not aborted. Conversely, 12.2% women indicated abortion across the setting. This distribution suggests that a slightly larger proportion of women remain not aborted (Fig. 2).

**Fig. 3:**
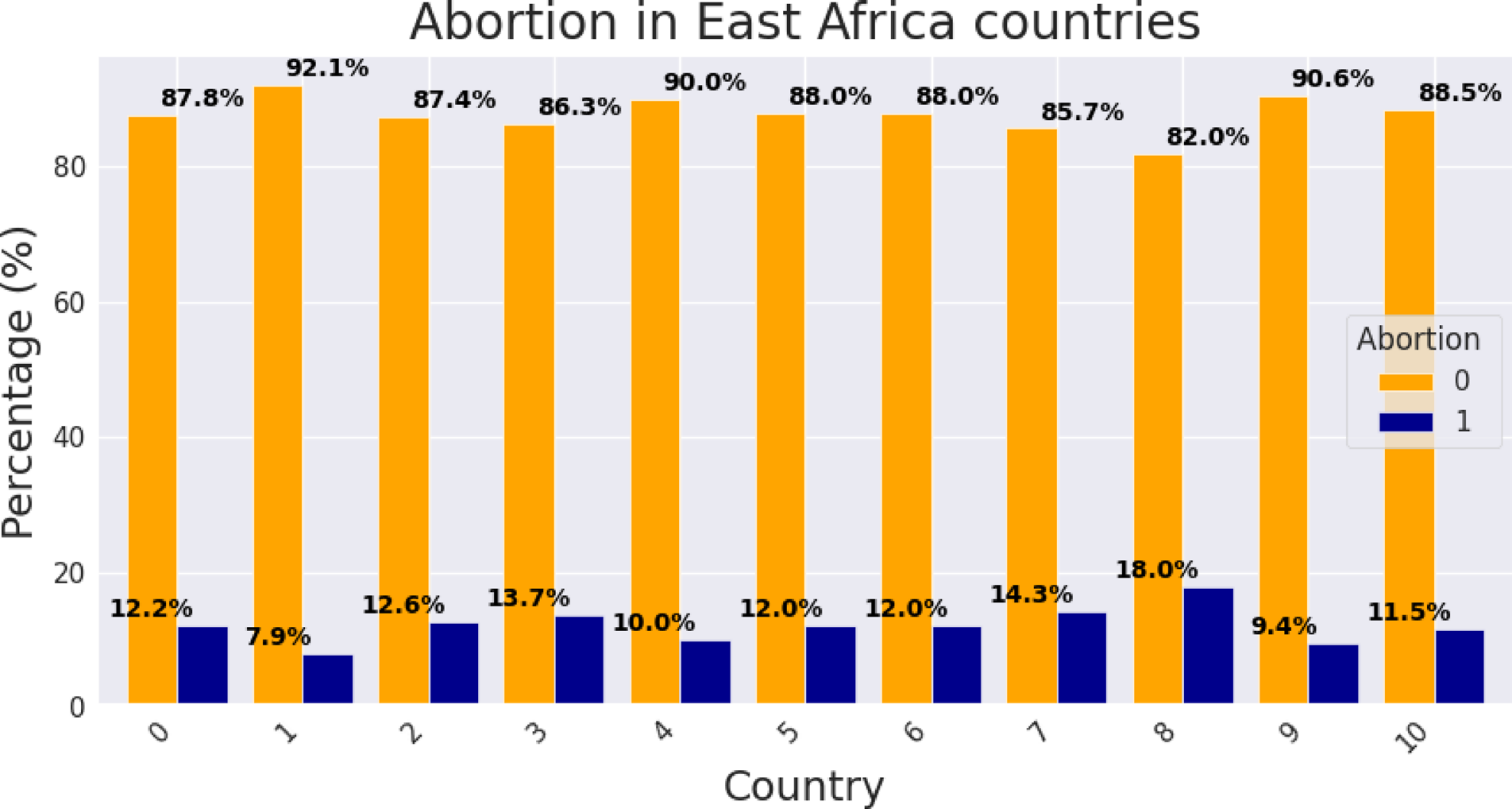
Distribution of abortion among reproductive age women across East African countries, 2015-2023GC. Country: 0 = Burundi, 1 = Ethiopia, 2 = Kenya, 3 = Madagascar, 4 = Malawi, 5 = Mozambique, 6 = Rwanda, 7 = Tanzania, 8 = Uganda, 9 = Zambia, 10 = Zimbabwe.

The bar chart below (Fig. 3) illustrates the percentage distribution of abortion across eleven countries, labeled 0 to 10. The abortion status was categorized to “0” and “1”, shown in orangey and dark blue bars respectively. Countries like Uganda, Tanzania, Madagascar, and Kenya have the highest proportion of abortion among women of reproductive age with percentage of 18.0%, 14.3%, 13.7% and 12.6% respectively.

### Machine learning analysis results

#### Data pre-processing results

In this study, the main pre-processing steps involved dealing with missing or null values, encoding categorical labels and checking for multicollinerty in the dataset. Due to the incompleteness of the initial dataset, missing values were handled using KNN. Input and output features must be numerical to be used with machines. This implies that to fit the dataset to the model, one hot encoder to encode categorical variables that are present in the dataset was applied. Thus, the one-hot encoding technique was used to address ordering problems. Each categorical value was translated into a new column with one-hot encoding, and the label values were created as new columns (1 or 0)^62^.

#### Model performance comparison

The performance of several machine learning models on unbalanced and balanced datasets is compared, and it is found that data balancing (using approaches such as SMOTE) often improves classification outcomes. Random forest outperformed the other models, with an accuracy of 0.82 to 0.86 and improvement of AUC from 74% to 91%. Decision tree and KNN followed closely after, retaining good AUC values of 88% and 79% respectively and improving accuracy after balancing. Overall, the results show that ensemble approaches, and that balancing the dataset improved accuracy across most models without significantly decreasing AUC, emphasizing the importance of dealing with class imbalance in predictive modeling (Table 3).

**Table 3:** Model comparison through tenfold cross-validation for all models evaluated on the test data.

Machine learning models	Performance	Unbalanced data	Balanced data
Dummy classifier	Accuracy (%)	0.45	0.49
	AUC	49	50
Random forest	Accuracy (%)	0.82	0.86
	AUC	74	91
Decision tree	Accuracy (%)	0.50	0.57
	AUC	54	88
KNN	Accuracy (%)	0.55	0.66
	AUC	59	79
XGboost	Accuracy (%)	0.43	0.69
	AUC	56	76
Logistic regression	Accuracy (%)	0.60	0.62
	AUC	69	70
Naïve bayes	Accuracy (%)	0.67	0.85
	AUC	56	69
Light boost	Accuracy (%)	0.62	0.73
	AUC	66	75
ANN	Accuracy (%)	0.70	0.89
	AUC	68	74
Adaboost	Accuracy (%)	0.72	0.86
	AUC	67	72
Catboost	Accuracy (%)	0.79	0.87
	AUC	67	76

**Fig. 4:**
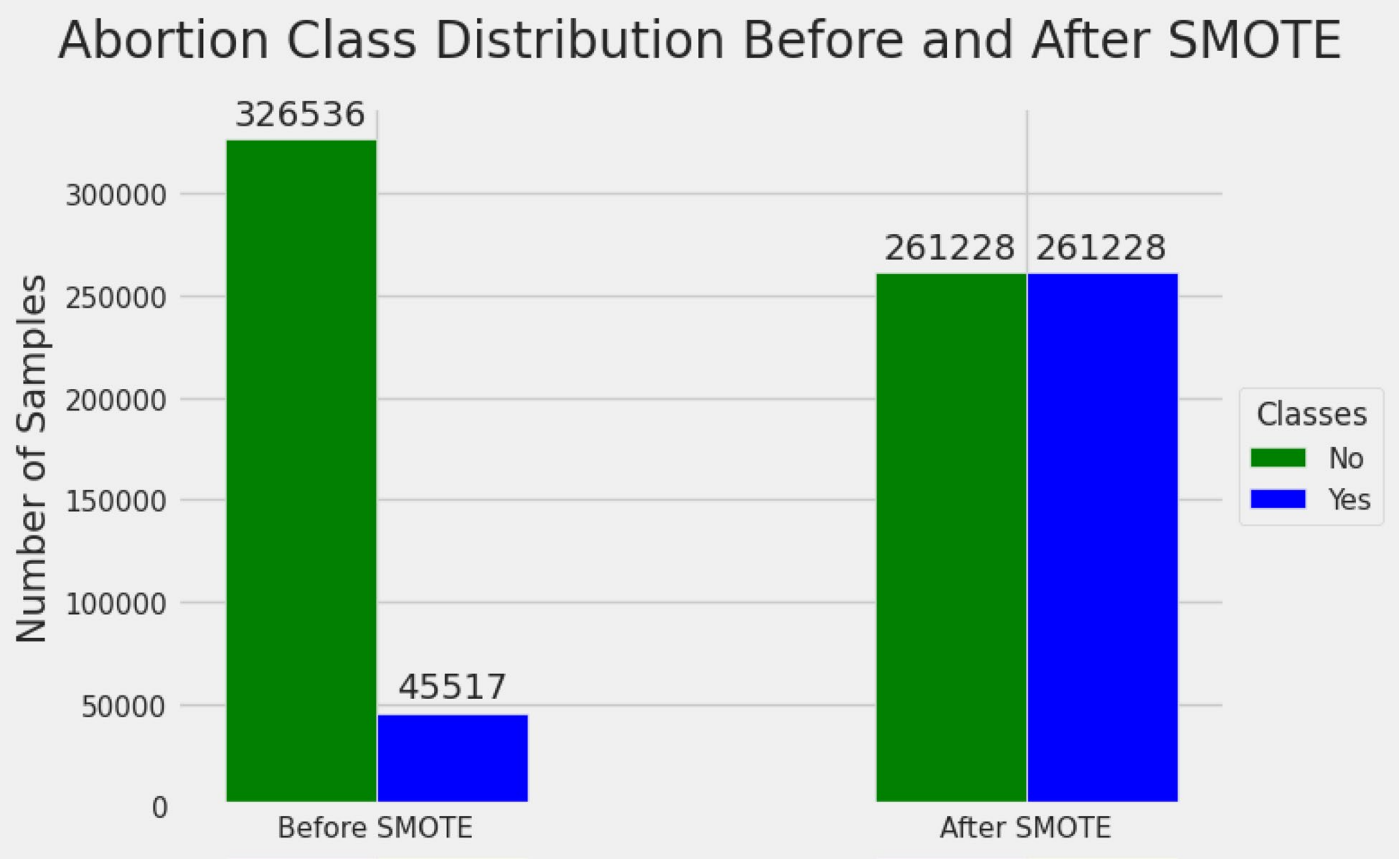
Distribution of abortion status before and after balancing using SMOTE.

The dataset showed a significant class imbalance between the two outcome categories before class balancing. At 326,536 samples in the majority class (“No”) and just 45,517 in the minority class (“Yes”), the minority class accounted for about 12.2% among the total observations. Machine learning models may become less sensitive at identifying instances of the minority class as a result of this imbalance. This issue was addressed by using SMOTE. The minority class was artificially increased to match the size of the majority group after oversampling. As a result, there were 261,228 samples in each class, creating a balanced dataset with a 1:1 class ratio. It is anticipated that this balancing process will reduce majority-class dominance during training, improving the model’s capacity to learn decision boundaries for the minority class. Consequently, the post-SMOTE dataset offers a better foundation for training classification models, especially when the goal of the study is to precisely identify minority outcomes (Fig. 4).

**Fig. 5:**
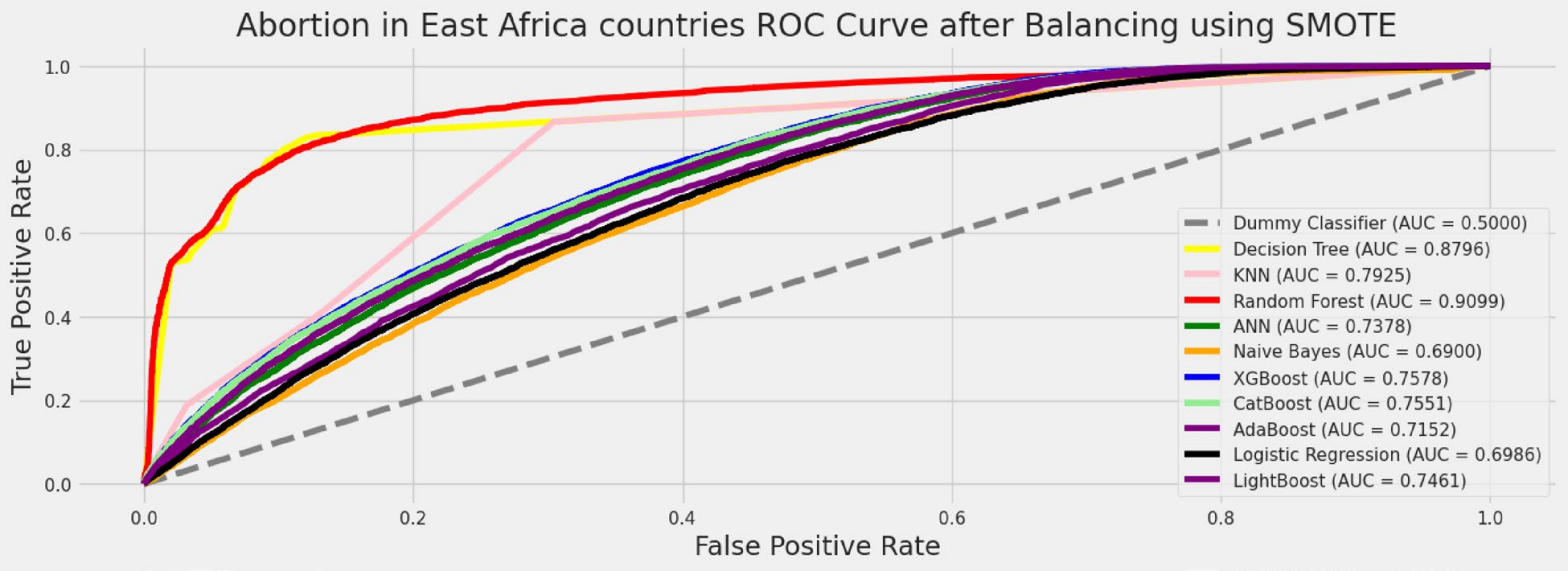
Comparison area under ROC curve on 20% test split for all models.

The ROC curve shown in the Fig. 5 compare the classification performance of eleven different supervised machine learning models used to predict abortion among women of reproductive age in East African countries.it plots true positive rate against false positive rate at various thresholds setting for each model. Accordingly, random forest achieved the highest AUC values of 0.91, indicating excellent classification performance.decision tree followed closely with AUC values of 0.88. KNN and XGBoost also performed well with AUC score of 0.79 and 0.76 respectively. The dashed diagonal line represent AUC = 0.5, which serve as a baseline. All models performed better than random with ensemble based models like random forest and. Decision tree demonstrating superior performance (Fig. 5).

### Random forest model performance

This experiment was developed to evaluate how well different classifiers predicted abortion. The objective of this analysis was to evaluate the accuracy of the predictions provided by the selected classifier. From the dataset, the random forest classifier’s performance was quite strong compared to other selected classifiers.

### Confusion matrix results

**Fig. 6:**
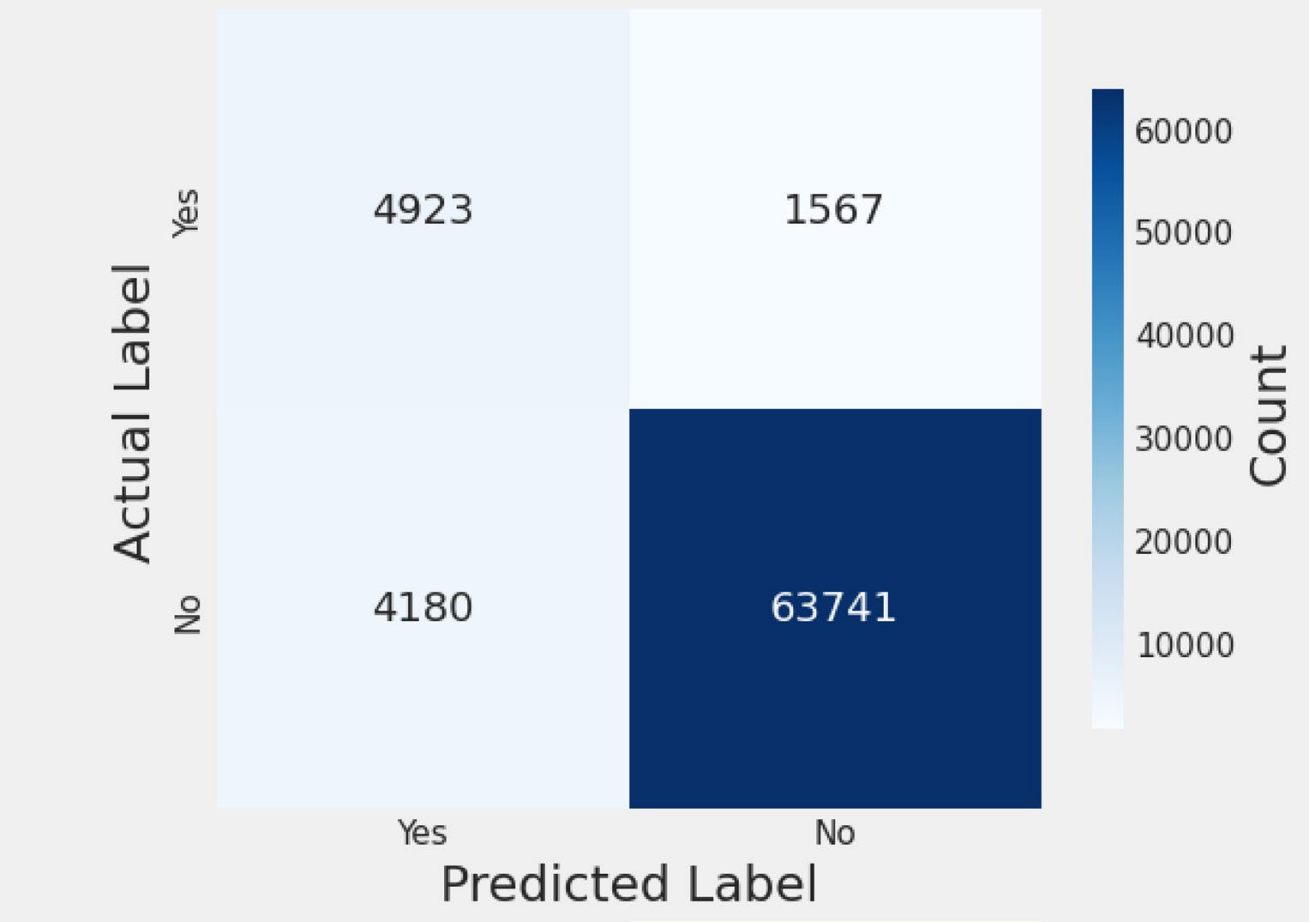
Confusion matrix of random forest classifier to predict abortion among women of reproductive age in East Africa countries, 2015-2023GC.

According to the confusion matrix, which is shown in Fig. 6 below, the model was correctly classified as true positive and true negative, which suggests that datasets outperformed for model building. As it indicates random forest demonstrates strong classification ability, correctly identify the majority of both yes and no cases. The number of true positive and true negative was high, indicating good performance in good sensitivity and specificity.

### Model interpretation/explanation

**Fig. 7:**
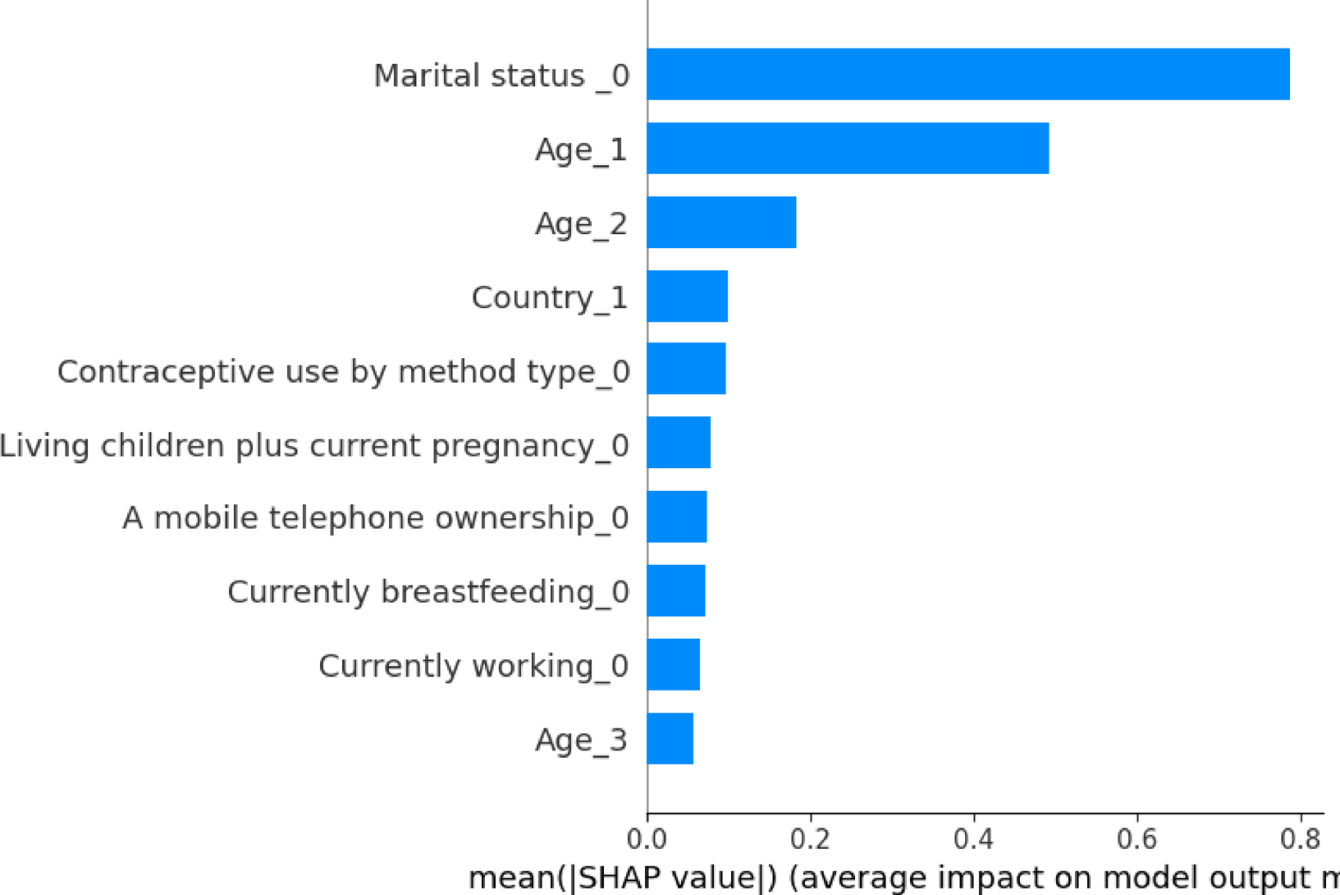
SHAP global feature importance plot of optimized random forest model. Marital status_0 (never in union), Age_1 (women those with the age of 15–19 years), Age_2 (women those with the age of 20–24 years), Country_1 (women from Ethiopia), Contraceptive use by method type_0 (women who not used any method), Living children plus current pregnancy_0 (women who have no living children plus current pregnancy), A mobile telephon ownership_0 (not own a mobile telephone), Current breastfeeding_0 (not breastfeeding currently), Currently working_0 (not working currently), Age_3 (women those with the age of 25–29 years).

This study has used model SHAP global feature importance for selecting top predictors of abortion among women of reproductive age in East African countries. This technique assesses the mean absolute SHAP value for each predictor over all data points to quantify the feature’s contribution to the predicted abortion. The predictors are ranked in descending order depending on their impact on the outcome variable prediction. Features with higher mean absolute SHAP values are more influential. Among the features, marital status had the highest average SHAP value, indicating that it was the most influential component in the prediction model. This was followed by age indicating significant predictor of abortion. Other relevant features included country, contraceptive use by method type, living children plus current pregnancy, a mobile telephon ownership, current breastfeeding, and currently working though with a lower impact (Fig. 7).

### Global predictors of abortion

**Fig. 8:**
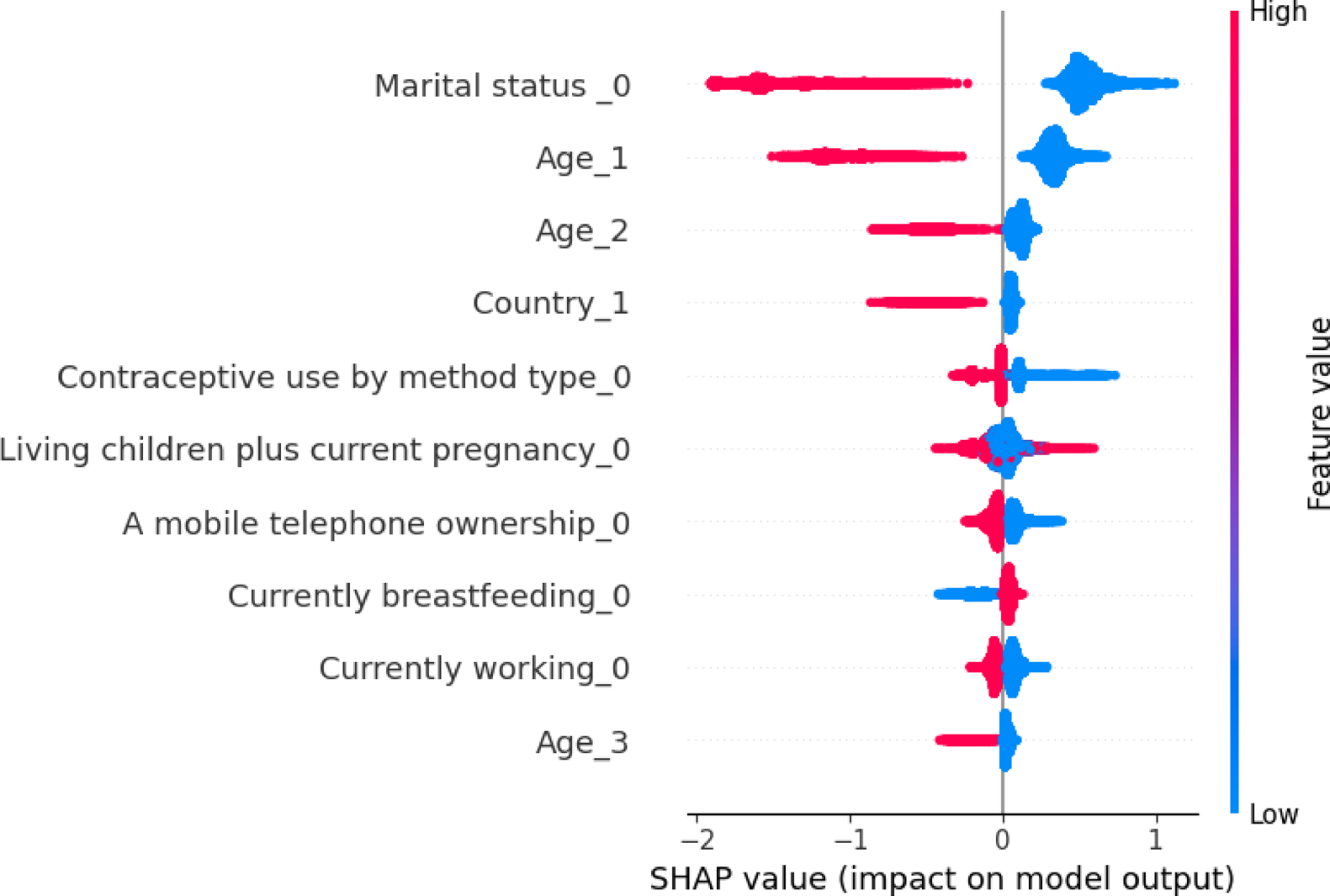
Beeswarm plot, ranked by mean absolute SHAP value generated by optimized random forest model. Marital status_0 (never in union), Age_1 (women those with the age of 15–19 years), Age_2 (women those with the age of 20–24 years), Country_1 (women from Ethiopia), Contraceptive use by method type_0 (women who not used any method), Living children plus current pregnancy_0 (women who have no living children plus current pregnancy), A mobile telephon ownership_0 (not own a mobile telephone), Current breastfeeding_0 (not breastfeeding currently), Currently working_0 (not working currently), Age_3 (women those with the age of 25–29 years).

SHAP Beeswarms plots were employed to present a comprehensive and clear overview of how the factors influence the model’s predictions across data sets. Figure 8 presents the distribution of the effects of each predictor on the model’s output (i.e. abortion) by plotting value of that particular predictor for every sample. The x-axis represents Shapley values of each variable on the model output computed from random forest model. Each row of the dataset represents its unique point for each variable. The points are arranged horizontally along the x-axis based on their SHAP value. Examining how SHAP values are distributed illustrates how a variable affects model predictions. The points on this plot reflect the Shapley values of the features associated to abortion, providing information about the relevance and relationship of each feature with the outcome variable. High/positive SHAP values indicate an increase in the anticipated probability, whereas low/negative SHAP values indicate a decrease in the predicted probability. As a result, points on the right side of the vertical line (0 SHAP value) increase the possibility to abortion, whereas points on the left side decrease the likelihood. The red and blue colors in the figure represent the greater and lower values of each predictor’s variables respectively. Hence.

Women whose marital status is never in union, women those with the age of 15–19 years, women those with the age of 20–24 years, women from Ethiopia, women who not used any method, not own a mobile telephone are less likely to about their pregnancy. While, women with no living children plus current pregnancy and not breastfeeding currently are more likely to abortion.

### Local predictors of abortion

**Fig. 9:**
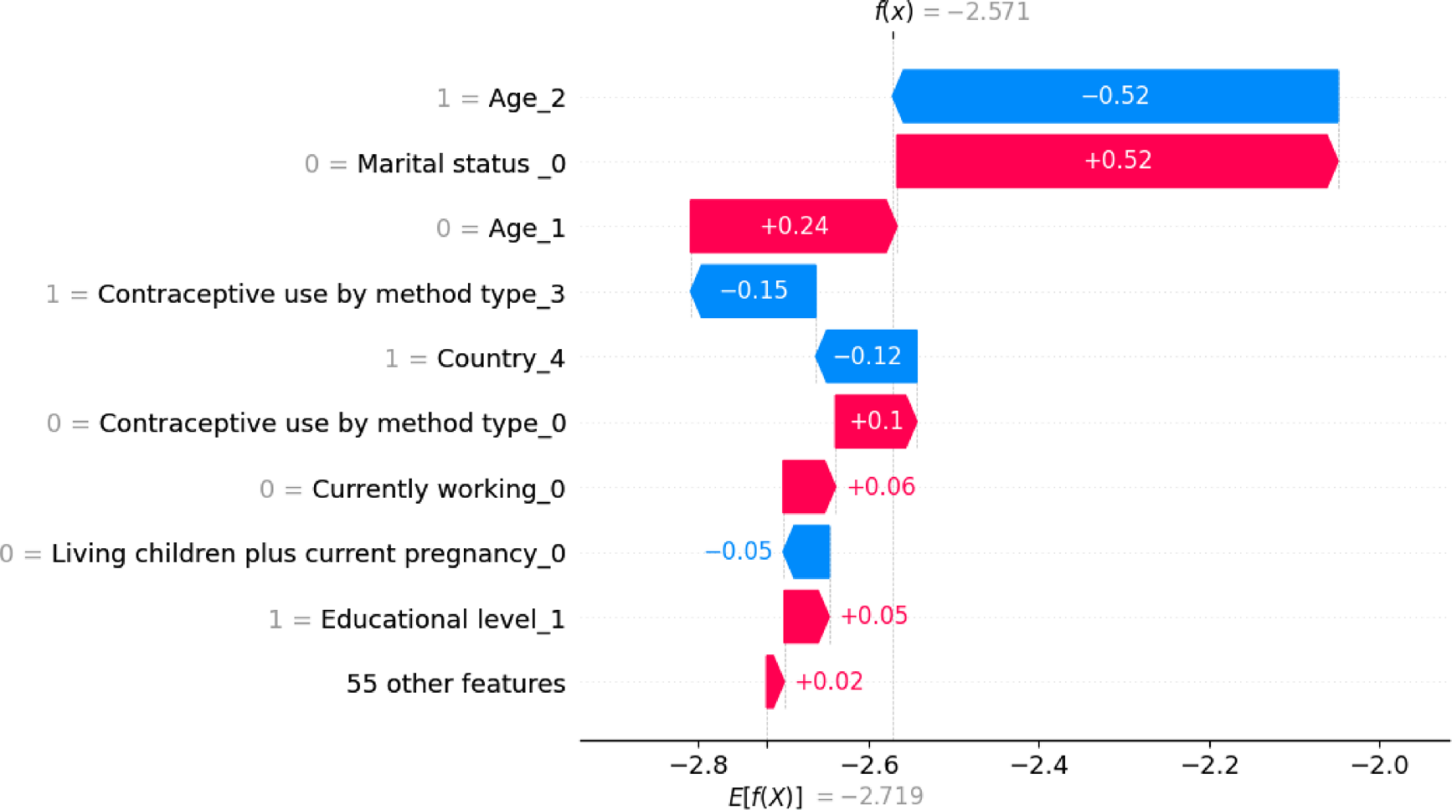
Waterfall plot displaying prediction of abortion on the first observation. 1=Age_2 (women aged from 20 to 24 years), 0 = Marital status_0 (women not never in union), 0=Age_1 (women not aged from 15 to 19 years), 1 = Contraceptive use by method type_3 (women who used moderns method), 1=Country_4 (those women from Malawi), 0 = Contraceptive use by method type_0 (women who used any method), 0 = Currently working_0 (women who working currently), 0 = Living children plus current pregnancy_0 (women who have living children plus current pregnancy), 1 = Educational level_1 (women with primary educational level).

Finally, waterfall plots were used to explain the model prediction of the first observations in Fig. 9. The waterfall plot begins with the expected value of the model output on the x-axis (E [f(x)] =-2.719), which reflects the starting prediction for the given sample before taking consideration of any feature contributions. This baseline prediction is usually the average or most common prediction in the dataset. For a particular observation, a model output above this value (E [f(x)]) correlates to a positive class (i.e., abortion), while scores below this value corresponds to a negative class. Hence for this observation, the combination the positive contributions (in red) and the negative contributions (in blue) moves the expected value output to the final model output (f(x) = 2.571) classified as positive class. Accordingly, women not never in union, women not aged from 15 to 19 years, women who used any method, women who working currently, and women with primary educational level drives up the probability for abortion. Whereas women aged from 20 to 24 years, women who used modern method, those women from Malawi, and women who have living children plus current pregnancy drives down the probability of abortion.

## Discussion

This study aimed to assess the prevalence and associated factors of abortion in East African countries using recent DHS data of East African countries. According to the findings of this study, abortion was found to be a significant public health problem in East Africa. The prevalence of abortion in this study was 12.2% is in line with studies conducted in Ghana, Mozambique, and Ethiopia^17,63–65^ and higher than the study conducted in selected East African countries^20,21^. Inadequate coverage and access to family planning services, greater rates of unplanned pregnancies, and higher rates of acute and chronic malnutrition among women of reproductive age could all contribute to East Africa’s increased abortion burden^66–68^. Unsafe abortion rates are also increased by restrictive abortion regulations and inadequate healthcare infrastructure^5,21^.

Following the selection of sixteen variables from the literature, the SHAP was used to determine the top ten features. An imbalanced dataset of training samples was used to train the classifiers. The outcome showed that the balanced accuracy of the prediction was low. As a result, we realized that data balancing significantly affects the classifier’s prediction performance, which is why the researchers utilize the resampling technique for data balancing. Lastly, the SMOTE sampling approach was used.

We understand that all eleven models in the abortion prediction analysis model have approximate prediction accuracy in data imbalance. However, the random forest model outperformed the other in terms of balanced accuracy and AUC in the balanced data set, indicating that it is a good classifier model. With a performance evaluation of 82% accuracy and 74% AUC in imbalanced train data and 86% accuracy and 91% AUC on balanced (SMOTE) data, the random forest was the best predictor model in this study. This was lower than the other study conducted in Ethiopia^69^. The possible difference might be due to the number of features used. The features could have an impact on how well the predictive model performs^46^.

According to the SHAP beeswarm and bar plots, marital status emerged as strong significant predictor of abortion. Women whose marital status were never in union are less likely to experience abortion. This finding is supported by the other studies conducted in sub Saharan Africa^21^. The possible explanation for the association between marital status and abortion could be the lack of contraceptive use or contraceptive failure^70^ In addition, research has shown that married women utilize abortion to restrict the number of children they have and to space out their pregnancies^71^.

The finding from this study shows that women with the age of 20–24 years are less likely to experience abortion, this is contrary with studies done in united states of America (USA)^72^, Denmark^73^ and 27 SSA countries^74^. Due to maternal health issues and conception-related illnesses such preeclampsia, ectopic pregnancy, and gestational diabetes, older women are more likely to have a high-risk pregnancy that could end in abortions^75, 76^. The fact that women in this age range are frequently in the early stages of their reproductive lives and may be more inclined to want children could be one reason for this observation. Pregnancies that occur around this time may therefore be more likely to be planned and, hence, less likely to terminate in the end.

Compared to women from other nations included in the study, Ethiopian women were found to be less likely to experience abortion. This result might be due to variations in legal frameworks, societal norms, access to reproductive health care, and the use of contraceptives among countries. Strong religious convictions, societal attitude associated with abortion, and cultural views around pregnancy termination may discourage Ethiopian women from obtaining or disclosing abortion services. The observed discrepancies may also be influenced by differences in country reproductive health legislation and the accessibility of safe abortion and post-abortion care services. Even when rates of unwanted pregnancies are still high, prior research has shown that restrictive social settings and restricted provider accessibility can lower the reported prevalence of abortion.

In this stuy, the other important predictor of abortion is the use of contraceptives by method type. Women who not use any contraceptives method are less likely to experience abortion, this contradict with the previous studies^20,21,77^. Given that not using contraception is typically linked to a higher risk of unplanned pregnancy and, thus, a higher likelihood of pregnancy termination, this conclusion may seem paradoxical. Furthermore, women who utilize contraceptive methods may be more informed of their alternatives, including pregnancy termination services, and are frequently more involved with reproductive health services. On the other hand, because abortion is discouraged by societal, religious, or personal convictions, women who do not utilize contraception may be more willing to accept pregnancy when it happens.This finding would have been supported by the fact that modern contraceptive users were less likely than traditional users to become pregnant unintentionally, which is usually terminated before the fetus reaches the age of viability^77,78^. This finding may be explained by the fact that traditional contraception users had a higher risk of unwanted or unexpected pregnancy, which is typically terminated before the fetus reaches the age of viability, whereas modern contraceptive users had a lower risk^79^.

Women who have no living children plus current pregnancy less likely to experience abortion. This contradict with findings from previous studies^20,80^. This could be explained by the fact that mothers with more parity may use maternal health services like family planning and have a greater understanding of menstrual cycles. These mothers could also know that the best strategy to limit the number of children and lengthen the intervals between births is to use contraception^21^. Additionally, this finding could be explained by the possibility that women who are childless have a greater desire to start a family and are hence more likely to carry a pregnancy to term rather than end it.

Generally, this study performed different machine learning classifiers, which are different from the statistical study. According to the study findings, machine learning classifiers could be a useful tool and the best data science approach that can produce high prediction accuracy and reliability in the prediction of public health studies. In addition, this study tried to compare the machine learning classifier with the statistical finding of the predictor of abortion.

### Strength and limitation of the study

This study’s primary strength were the use of large sample sizes, nationally representative data, and the use of advanced statistical method, specifically a machine learning approach, which revealed previously undiscovered relationships and patterns. To determine the relative significance of each predictor and obtain an understanding of how each component contributed to the model’s predictions, this study employed a variety of methodologies, including SHAP. This helped to comprehend how various factors affected the model’s predictions. Moreover, the application of SHAP values provided a robust framework for understanding feature importance, offering nuanced insights into how various factors influence abortion. The focus on a relevant and rich dataset, coupled with the application of advanced analytical techniques and comparisons, make this study as a significant addition to the literature on abortion, informing policymakers and practitioners about critical intervention points to reduce the impacts of abortion in the region.

Despite the valuable insights gained from this study, a few limitations must be acknowledged. First, the reliance on secondary data from the DHS may restrict the depth of analysis regarding the causal relationships between the identified features and abortion. While DHS datasets are comprehensive and nationally representative, they are inherently cross-sectional, capturing a snapshot of health behaviors at a single point in time. This limitation constrains the ability to assess temporal dynamics or changes in target variable, which could vary significantly due to interventions, policy changes, or evolving socioeconomic conditions. Additionally, self-reported measures within the dataset may introduce biases related to recall or social desirability, particularly in sensitive areas such as healthcare access and maternal education. Such biases could obscure the true extent of the relationships between variables, potentially impacting the reliability of the findings. Future studies could benefit from exploring additional predictive variables that can be identified.

## Conclusion

Although maternal mortality has declined in East Africa in recent years, abortion remains a major public health concern. The Beeswarm plot of the SHAP analysis based on the random forest model showed that marital status, age, country, Contraceptive use by method type, Living children plus current pregnancy, A mobile telephon ownership, Current breastfeeding, and Currently working status were the top predictors of abortion. Therefore, throughout the intervention, it is preferable to take into account the high-risk groups in order to prevent abortion among women of reproductive age.

## Data Availability

The data for this study were obtained from the DHS Program. These datasets are publicly available and can be requested from the DHS website at http://www.dhsprogram.com.

## Abbreviations

AUC: Area under the ROC curve
DHS: Demographic and health surveys
SMOTE: Synthetic minority over-sampling technique
